# Blocking the Law from Within: Familyism Ideologies as Obstacles to Legal Protections for Women in El Salvador, Guatemala, Honduras, and Nicaragua

**DOI:** 10.1017/lar.2022.72

**Published:** 2022-12-06

**Authors:** Cecilia Menjívar, Leydy Diossa-Jiménez

**Affiliations:** Department of Sociology, UCLA, Los Angeles, California, USA

**Keywords:** Central America, symbolic violence, violence against women laws, judicial systems, familyism, gender violence

## Abstract

The scholarship seeking to explain the ineffectiveness of violence against women (VAW) laws has focused on the lack of resources or will to implement these laws. Less attention has been given to how these laws are crafted and positioned in the legal hierarchy, which may undermine them from the start. This article focuses on four cases from Central America, a region where fifty-five laws to protect women from violence were passed between 1960 and 2018, yet VAW continues. It finds that the legal positioning and language of these laws prioritize family unity while undermining women’s rights to protection; thus, these laws fail by design. The article identifies four legal placements that delay (El Salvador), undermine (Honduras), diminish (Nicaragua), or render abstract (Guatemala) the effectiveness of VAW laws in the context of penal and judicial codes. This work has direct policy implications and broader relevance beyond the cases examined here.

## Resumen

Los estudios sobre la ineficacia de las leyes de violencia contra las mujeres (VCM) se centran en la falta de recursos o voluntad para implementarlas. Menos atención han recibido su elaboración y posicionamiento en la jerarquía legal, lo que puede socavarlas desde su origen. Este artículo se centra en cuatro casos de Centro América, una región donde se aprobaron cincuenta y cinco leyes para proteger a las mujeres entre 1960 y 2018, sin embargo, la VCM continúa. El posicionamiento legal y el lenguaje de estas leyes priorizan la unidad familiar socavando los derechos de las mujeres a la protección; por lo tanto, estas leyes fallan por diseño. El artículo identifica cuatro emplazamientos legales que retrasan (El Salvador), socavan (Honduras), disminuyen (Nicaragua) o abstraen (Guatemala) la efectividad de las leyes de VCM en el contexto de los códigos penal y judicial. Este trabajo tiene implicaciones para políticas públicas y una relevancia más allá de los casos examinados aquí.

Honduras, El Salvador, and Guatemala often top the list of countries with the highest levels of feminicide in Latin America and the world.^1^ In 2019, Honduras had the highest feminicide rate in the region at 6.2 per 100,000, followed by El Salvador with 3.3 and Guatemala with 1.8 (CEPAL 2021). What accounts for these rates, despite multiple laws on the books and efforts to protect women? We argue that despite these countries’ numerous laws addressing violence against women (hereafter VAW) and codifying feminicide as a crime, entrenched gendered ideologies prioritize the family as the fundamental unit of society and disadvantage women. We examine how familyism ideologies^2^ permeate these countries’ legal systems to undermine the language and positioning of VAW laws vis-à-vis the penal and judicial codes, limiting the potential of VAW laws.^3^

Although existing scholarship primarily focuses on violence against women in El Salvador, Guatemala, and Honduras, we include Nicaragua to strengthen the scope of our analysis. In all four countries, familyism ideologies reemerged in the post-conflict context of the 1990s, when conservative political and religious groups found common ground in promoting such policies as national unity narratives (O’Brien and Walsh 2020). Although revolutionary Nicaragua had comparatively more progressive VAW laws, the recent conservative movement has led to a reversal of many gains. The analytical inclusion of Nicaragua reveals the crucial place of the emergence of familyism, which has undermined VAW laws in the region (Chant 2003). Each case we examine contributes to our explanation as each exemplifies a form of familyism ideology shaping the content and legal positioning of VAW laws and undermining their power.

Between 1960 and 2018, El Salvador, Guatemala, Honduras, and Nicaragua approved fifty-five laws^4^ to prevent and prosecute violence against women, yet these laws have not translated into safer conditions for women. For instance, despite approving twenty-six laws during this period, Guatemala had 2,898 violent deaths of women and feminicides between 2016 and 2021 (GGM 2022). Despite having ten and twelve laws on the books, respectively, El Salvador reported 1,340 feminicides and Honduras reported 2,249 feminicides between 2013 and 2020 (CEPAL 2022). Nicaragua has seven laws to protect women on the books and reported 174 feminicide cases from 2012 to 2018.^5^ Despite the widespread approval of legal provisions in these Central American countries, however, such laws have remained ineffective.

We thus examine how familyism ideology, which we examine as a form of symbolic violence, is reflected in VAW laws and refracted in different positionings and misalignments between VAW laws and the judicial and penal codes. These positionings mirror unequal gender imbalances in society at large and amplify and reproduce inequalities that disfavor women within the justice system itself. Such positionings delay, undermine, diminish, or render abstract the effectiveness of VAW laws in relation to the penal and judicial codes. From their inception to their inferior juridical positioning (and the language of their content), VAW laws are diluted; therefore they fail by design.

Gender ideologies and orthodox family values that uphold family unity at any cost permeate the language, content, and legal positioning of VAW laws, placing women in deeply unequal positions that diminish their lives and rights as citizens. As Velásquez Díaz, Vargas Quintanilla, and Pérez Villanueva (2016, 74) observe, a significant form of sexism manifested in law is familyism: “it is the idea that women and family are synonyms and therefore their needs and interests are the same. Family is taken as a whole, as a unit, where the realities of each of its members are irrelevant. This form of sexism is frequently used among administrators of justice when, regardless of circumstances, women are encouraged to return to their aggressors, forgive them, give them a second chance, think about their family and the men, and do it for their children.”

Structural inequalities that amplify gender imbalances and attitudes toward violence in women’s lives contribute to normalizing the mistreatment of women through individual acts and by legal and judicial institutions and society more generally (Menjívar 2011). Simultaneously, inadequacies in implementing VAW laws reinforce and reproduce the naturalization of women’s disadvantaged position: the symbolic violence that comes from the message that their lives do not matter as much as their families. In contexts of widespread gender violence toward women, such as in Central America (Menjívar and Walsh 2017; Musalo and Bookey 2013; Walsh and Menjívar 2016), the ineffective implementation of VAW laws fails to challenge violent practices and contributes to normalizing and reproducing them, underscoring the routine symbolic violence in women’s lives. Following Bourdieu (2001), we suggest that the faulty implementation of VAW laws in Central American countries renders unproblematic (and hence legitimate) the systematic exercise of gendered violence by individuals and institutions.

Based on our analysis, we suggest a typology of the legal positioning of VAW laws within a broader legal landscape (vis-à-vis the penal and judicial codes) to highlight the privileged position of familyism over women’s lives that VAW laws reflect. Our analysis of VAW laws’ language and legal positionings in the four countries we examine goes beyond explaining gaps between laws on the books and laws in practice. It shows how the symbolic violence of familyism permeates VAW laws from their inception to their implementation. Each case we analyze exemplifies an obstacle to justice arising from VAW laws’ legal positioning that is salient in that country, though not exclusive to it. In each case, the articulation of laws explains, at least in part, the remarkable failure of the justice systems in the Central American region to protect women.^6^

## Inefficacy of VAW laws

The scholarship seeking to explain the lack of implementation of VAW laws can be summarized in three strands (Gould and Barclay 2012). One strand focuses on the legal doctrine (structure) of the law that makes it challenging to implement (Ngozi, Iyioha, and Durojaye 2018). This perspective suggests that the laws’ evidentiary standards are obstacles to its implementation (Adamson, Menjívar, and Walsh 2020).^7^ When VAW laws are held to standards of other laws (i.e., penal code), providing evidence presents a challenge because cases of rape and feminicide often lack the evidence necessary to prove that the perpetrator committed the crime (England 2018). A second strand focuses on state actors’ or agents’ lack of will to implement laws. Ghosh and Choudhuri (2011) explain the continued high rates of domestic violence and feminicides in India despite a comprehensive reform in 2005. Police officers’ apathy and disdain can undermine the law’s enforcement teeth (Medie 2015). In Honduras, for example, state agencies have diverted resources away from efforts to protect women from violence to address more “serious crimes” (e.g., burglaries or gang crimes), thus belittling violence against women (Menjívar and Walsh 2017). A third strand focuses on how “adjacent” laws governing marriage, family, and work (Adamson, Menjívar, and Walsh 2020; Menjívar and Walsh 2016) may undermine the enforcement of VAW laws on the ground. This literature suggests that women seeking justice face challenges when other laws (e.g., civil code) are prioritized and are endangered when, for instance, restraining orders are denied because these violate the property rights of the aggressor (e.g., property law).

Violence against women laws are embedded in legal systems that contribute to maintaining the gap between the approval and implementation of legal provisions. Relationships between VAW and “adjacent laws” (e.g., those governing marriage, alimony, work, and land tenure) contribute to reproducing the symbolic and structural violence that women experience daily (Adamson, Menjívar, and Walsh 2020; Walsh and Menjívar 2016). The scholarship on failures of VAW laws examines the importance of these links for protecting or undermining women’s rights (Gould and Barclay 2012). However, less attention has been given to factors within the legal structure that may hinder the implementation of VAW laws. Thus, rather than examining our cases through a “gap” lens (i.e., between law on the books and law in practice), we focus on the positioning of VAW laws within the legal context and the language used to craft such laws, which together undermine women’s plight, diminish their protection and constitutional right to equity, and formally reproduce gender inequalities. Our argument goes beyond legal dimensions to unveil familyism ideologies that curtail justice for women in situations of violence.

Building on prior scholarship, we examine what happens before implementation, at the laws’ origin. We focus on the familyism language embedded in VAW laws and the inferior positioning of VAW laws within the normative hierarchy.^8^ Our contribution unveils obstacles within normative legal hierarchies that later, at implementation, render VAW laws inefficient and challenging to implement. We focus on four legal positionings—undermined sanctions, de-scaled implementation, abstracted standardization, and delayed execution—all predicated on “familyism” ideologies inscribed in VAW laws.

## Theoretical framework

We situate VAW laws’ legal positionings vis-à-vis the penal and judicial codes and conceptualize the relationship between these laws as expressions of the symbolic violence embedded in familyism ideologies. From this standpoint, VAW laws’ alignment within the legal landscape constitutes an obstacle in women’s search for protection and justice.^9^ Following Bourdieu and Wacquant (1992, 171–173), we argue that gender domination operates through expressions of symbolic violence that render the “male order” as self-evident, taken for granted, and beyond the need for explicit justification—as “natural.”^10^ This gentle and less visible violence (because it stops short of physical injuries) takes different forms such as androcentricity, gender insensitivity, and familyism (Eichler 1988). The naturalization of the male order contributes to reproducing a shared sense of gender relations in which women are misrecognized as objects in male-dominated circuits of exchange (e.g., matrimonial markets) (Bourdieu 2001, 42–43). The physical violence that transforms women into objects of abuse is an instance—and often the ultimate consequence—of pre-existing forms of symbolic domination.

The legislative and judicial branches of the state play a crucial role in inscribing the fundamental “principles of the androcentric vision” in the rules that define the official status of citizenship (Bourdieu 2001, 87). Masculine domination, therefore, operates at the level of subjective experiences and interpersonal relationships (Menjívar 2011) and in routine practices of formal and informal political institutions (Mackay, Kenny, and Chappell 2010, 580–582). The rules of the juridical game are embedded in symbolically prescribed forms of masculinity and femininity and orthodox ideas of the family, which operate as gendered assumptions to act within the state’s domain. In this sense, presidential decrees, congressional legislation, and judicial rulings can be conceptualized as political outcomes shaped by gendered dispositions informing concrete practices within the state. Thus, the state is the preeminent institution that legitimately imposes principles of vision and division of the social world, including those governing gender (Bourdieu 2000, 186). A homology is thus created between the dominant vision of the world—that is, the gendered division of the world—and the legal orthodoxy guaranteed by the state through its legal practices.

How does familyism, as symbolic violence, permeate the VAW laws of El Salvador, Guatemala, Nicaragua, and Honduras? How does the inferior legal positioning of VAW laws in relation to the penal and judicial codes create various obstacles that reproduce and reinforce women’s lesser position in society? The answer lies in a critical feedback loop: the devalued position of women is naturalized and reproduced in the language of laws and reflected in the laws’ lower positioning in the legal hierarchy, which then reproduces and solidifies gendered conceptions and violence in society. This is buttressed by familyism, the ideology that defines women as primary household caregivers and the family as superseding women’s needs. This conceptualization of the family is based on male authority and founded on unequal power relations between the man—normalized as the head of the household—and the rest of the family members (Facio 1992). Although familyism is based on tradition, its contemporary ideology presents a nuclear formation composed of legally married parents and their children as aspirational and universal to society (Bernardes 1985).^11^ Motherhood expectations are concomitant with dependence on and respect for the male spouse’s authority, which presumably assures the successful upbringing of children. In this scenario, women are responsible for the family’s well-being and care; any digression endangers the family. Women then do not exist as individual subjects or citizens with needs and rights.

The dominant, gendered conception of women shapes and is shaped by how new laws are incorporated into the legal apparatus, which does not escape familyism biases. The legal context adopts the dominant familyism ideology as normative and, in a feedback loop, formalizes women’s inferior position and naturalized views of their lower position in the family. As Bourdieu (1987, 842) suggests, “the ethos of legal practitioners … and the immanent logic of the legal texts … are strongly in harmony with the interests, values, and worldviews of these dominant forces.” Legal harmonization of existing and new laws produces a “normalization effect” that reinforces the power of gendered conceptions of social reality (Facio 1992). The process of codifying male-dominant viewpoints into official regulations (i.e., penal and judicial codes) contributes to “transmuting regularity (that which is done regularly) into rule (that which must be done), factual normalcy into legal normalcy, simple familial *fides* (trust) … into family law” (Bourdieu 1987, 846). The definition of women as family caregivers ensures legislation that undermines women’s efforts to seek protection as individual subjects (as citizens in their own right). In this way, law legislates women as “women-mothers” or child bearers and not as “women-persons,”^12^ keeping them from any deviations and rewarding them for their capacity to sacrifice and satisfy the family’s needs even when their safety is compromised (Menjívar 2011). In these instances, the gendered vision and division of the social world—foundational to gendered forms of violence—are intertwined with the exercise of juridical power.

## Country cases in context

The Central American countries in this study have experienced extended periods of violence. As Menjívar (2011) has shown, the political economy of violence has differential effects on women, deepening gendered power asymmetries and inequalities. Social class—a critical marker that shapes how people live (and die)—intersects in various ways with gender violence. For instance, women from higher social classes who are victims of domestic violence also experience a negligent legal system that preserves the status quo (e.g., the value of men’s rights over women’s lives). This social context has contributed to reinforcing gender inequalities by unevenly applying VAW laws by class, rurality, ethnicity, and place (Beck 2021). By handpicking cases in which to apply the law, legal systems in Guatemala, El Salvador, Nicaragua, and Honduras have contributed to the perpetuation (and normalization) of violence toward women. Therefore, in Central America, gender violence contexts expose women to differentiated forms of physical and nonphysical mistreatment that further diminish their life chances and understandings of the self (Menjívar 2011; Silber 2011). It is at this level where laws without real enforcement teeth shape what women think is possible or impossible in their lives.

The positioning and language of VAW laws are legal expressions of women’s devalued lives in society, both inside and outside the courtroom (Menjívar and Walsh 2016). In the four countries we examine, women confront structural inequalities that disadvantage them in the short and long term. For instance, according to the Gender Inequality Index in Latin America, the four countries in this study have the lowest scores in the region: El Salvador, Guatemala, Nicaragua, and Honduras ranked 124, 127, 128, and 132, respectively, out of 189 countries. While in Latin America the average maternal mortality rate in 2017 was 73 per 100,000 live births, the averages in El Salvador, Guatemala, and Honduras were 46, 95, and 65 deaths per 100,000 live births, with Nicaragua having the highest, at 98 per year. In 2019, only 45.3 percent of Salvadoran women (aged fifteen and older) participated in the formal labor force compared to 75.7 percent of men; female participation in the labor force in Guatemala was 39.9 percent of women compared to 86.3 percent of men, in Nicaragua, 49.7 percent of women compared to 84.2 percent of men, and in Honduras, 52 percent of women compared to 85.9 percent of men (UNDP 2020). These gender disadvantages manifest in the inferior positioning (and poor execution) of VAW laws; concomitantly, such laws become a powerful mechanism for the normalization (and reproduction) of inequalities and multiple forms of violence against women. Significantly, the four cases show that familyism takes precedence even in VAW laws. The legal positionings of VAW laws vis-à-vis penal and judicial codes and their familyism language become expressions of symbolic violence against women embedded in law.

## Data and methods

Our analysis begins in the 1970s, when penal codes shaping current VAW laws came into effect. We employ primary and secondary data including laws, decrees, court cases, newspaper articles, and reports. We use primary data, including twenty-eight VAW laws in El Salvador, Guatemala, Honduras, and Nicaragua, to examine how familyism permeates VAW laws’ language to undermine their positioning vis-à-vis the penal and judicial codes and weaken their implementation. We also reference forty-seven legal documents, including Supreme Court Sentences, penal and judicial codes, and analyze sixteen laws in the penal and judicial codes. Additionally, we draw on secondary data from newspapers, reports by civil society organizations, and country-level statistics on intrafamilial violence and feminicides. We also use statistical reports from women’s organizations in the region (e.g., Grupo Guatemalteco de Mujeres, Organización de Mujeres Salvadoreñas por la Paz). Triangulation of these data sources helped us to inductively develop a conceptual typology to understand the four obstacles in the four countries’ legal landscape.

We studied the context of production and the historical evolution of the laws. Following Facio and Eichler, we identified gender ideologies embedded in the laws’ text (e.g., androcentrism or sexual dichotomy), examining how women are described and defined in the laws (e.g., married, poor, pregnant) (Eichler 1988, 8), definitions that serve as the building block for the VAW and feminicide laws (i.e., women-mother, women-family, or women-person) (Facio 1992, 75–110). Finally, our empirical strategy is tied to our theoretical framework. Thus, the analytical structure we advance focuses on the language, positioning, and alignments between the VAW laws and penal and judicial codes in Guatemala, Honduras, El Salvador, and Nicaragua. Each country case illustrates one type of legal positioning that shows one modality through which VAW laws are hampered by their alignment or misalignment with penal and judicial codes, to render VAW laws ineffectual at their origin.

## VAW laws in legal context: Guatemala, El Salvador, Honduras, and Nicaragua

Between 1960 and 2018, Guatemala, El Salvador, Honduras, and Nicaragua approved fifty-five laws to protect women from domestic violence, gender discrimination, and feminicide.^13^ As early as 1950, these countries signed or ratified international conventions and women’s civil and political rights, and they all approved the Convention on the Elimination of all Forms of Discrimination against Women (CEDAW 1979). In 1981, Guatemala signed this agreement and approved its own law, and in the same year, El Salvador approved the convention via legislative decree, while Honduras signed it in 1980 and ratified it in 1983. Finally, Nicaragua also signed the convention in 1980 and ratified it in 1995. By 1995, all four countries had ratified the Inter-American Convention to Prevent, Punish, and Eradicate Violence against Women adopted in Belém do Pará in 1994 (OEA 1994).

Although Guatemala and Honduras developed judicial infrastructures, the misalignment between their judicial system and VAW laws weakens women’s protection. The misalignment between VAW laws and the penal code results in ambiguous (if not altogether absent) penal classification, with severe consequences for women seeking justice. While the penal code outlines the protocols for the prosecution of violent and nonviolent crimes (e.g., feminicide and domestic violence), the judicial code defines the court system’s structure, operation, jurisdiction, and competence. Whereas VAW laws in Honduras and El Salvador make little to no reference to the penal code, Nicaragua and Guatemala developed incongruent standards for VAW laws’ implementation. Additionally, the lack of significant reform of the judicial codes to properly incorporate VAW laws impedes the effective prosecution of cases. When misalignments are not rectified and judicial codes are left unchanged, VAW laws are hampered at their origin, even before they can be implemented.

The improper alignment or complete misalignment of VAW laws with these countries’ penal and judicial codes is not accidental; it results from familyism ideologies embedded in VAW laws from their inception. When VAW laws equate women’s rights with those of the mother and the family and not their rights as “women-individuals,” the jurisdiction of VAW laws is defined a priori as the jurisdiction of the family code.^14^

Since the mid-2000s, the primary objective of VAW laws has remained to protect women as individuals who, having been objects of gender violence, are subjects with rights. Therefore, domestic and familial violence cases have been historically handled under the jurisdiction of family courts. However, in a context of gender inequality that devalues women and their bodies, familyism permeates even these legislative initiatives and is refracted in four different forms. When women are defined as individuals in VAW laws, no agencies for prosecution are created (e.g., lack of gender-specialized courts), as exemplified in the case of Nicaragua, and cases are sent to family courts. Or high evidentiary standards are applied so that the cases have no foundation in specialized gender courts, as in the case of Guatemala. When women are defined as women-family, VAW laws are not in agreement with the penal and judicial codes, and cases remain in family courts, as exemplified in the case of Honduras. Alternatively, due to the lack of agreement between VAW laws and the penal codes, gender-specialized courts prioritize other laws to protect perpetrators, as in the case of El Salvador.

**Figure 1.**
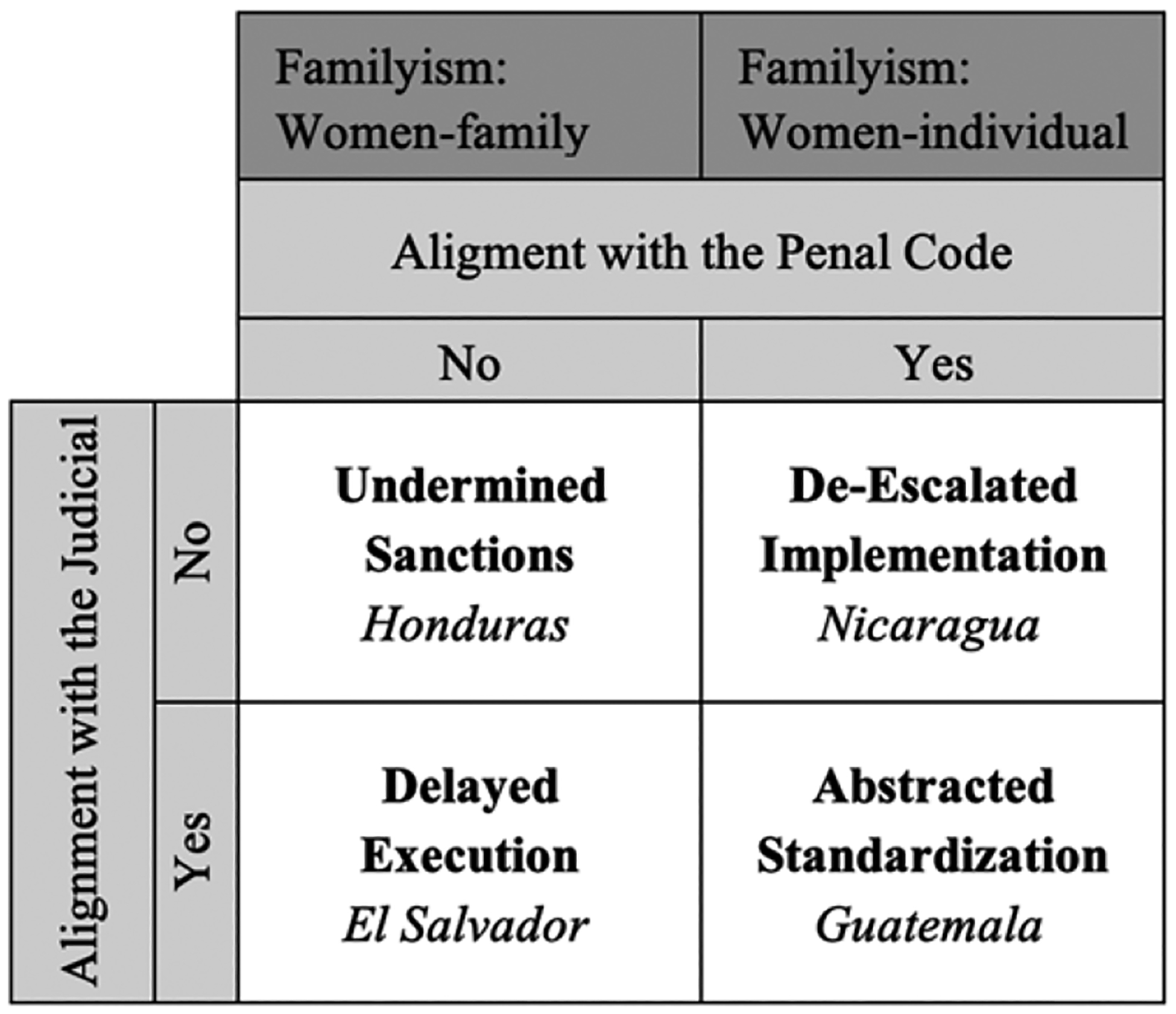
Alignment/misalignment of VAW laws with penal and judicial codes in Central America.

The different forms of misalignment between VAW laws and penal and judicial codes predicated on familyism ideologies (and language) create the legal conditions for the four unfavorable positionings for VAW laws we examine here (see Figure 1). Honduras represents a case of *undermined sanctions*, in which its penal and judicial codes are not in agreement with VAW laws. Nicaragua exemplifies a case of *de-escalated implementation*, in which VAW laws are aligned with the penal code, but the judicial code fails to provide specialized resources and procedures for prosecution. Guatemala represents a case of *abstracted standardization* of the law. Although VAW laws are congruent with the penal and judicial codes, violence against women and feminicide cases are held to the higher evidentiary standards required in penal and judicial codes, which are nearly impossible to meet given the nature of crimes against women. Finally, El Salvador constitutes a case of *delayed execution*. Due to the misalignment between VAW laws and the penal code, perpetrators of domestic violence or feminicide invoke other laws to postpone their sentences, arguing that VAW laws violate their right to due process and presumption of innocence, despite the creation of specialized prosecution units.

## Familyism and the unfavorable positioning of VAW laws in the legal system

### Undermined sanctions: The case of Honduras

The first legal positioning that diminishes the implementation of VAW laws is undermined sanctions, in which the penal and judicial codes are not in agreement with the content of VAW laws and familyism ideologies inform the content of these laws. This misalignment leads to few or no sanctions for those who violate VAW laws, which means that few cases are reported and an even smaller share of those reported are resolved. Furthermore, punishments in these cases provide symbolic, not substantive, reparations to the victims (e.g., community service). Thus, the punishment adjudicated for violence against women(i.e., up to three months of community service) is meaningless in relation to the severity of the crimes.

Honduras has approved two laws to protect women from domestic violence. The first, Decree 132 of 1997 (Law against Domestic Violence) aimed to protect the family, marriage, maternity, and childhood.^15^ The law described different forms of domestic violence (e.g., physical, psychological, patrimonial, or sexual) and provided protection mechanisms such as restraining orders and temporary separation of the aggressor from the victim’s home. Nevertheless, the principles of the law were undermined by its chapter on sanctions, and one to three months of community service became a symbolic reparation, replacing proportional punishments. The law’s main objective was to protect the family, and women were equally at risk of being charged with domestic violence even in cases where they were the victims.

In 2005, Honduras reformed the domestic violence law (Decree 132) with Decree 250.^16^ It declared the state’s responsibility to protect family and women, equating women’s needs with family’s needs. This reform was a significant setback within the already weak VAW law of 1997. Decree 250 reduced the months of community service for first offenses and for noncompliance with the protections of the 1997 VAW law from three years to one to three months in prison. It diminished the penalty on recidivism cases by modifying charges and penalties, framing them as disobedience to authority, lowering the punishment from four years to a maximum of three years in prison. This reform included the penalization of women for domestic violence. If women act in self-defense in domestic violence cases, safety measures are applied to both family members, including restraining orders to both parties and detention (Article 10). Following familyism ideologies, this change treated all family members equally even though women are the preeminent targets of domestic violence, protected family unity, solidified a hierarchy where women were not a priority for protection, and neglected women’s needs and rights.

In 2013, Honduras approved its feminicide law and defined it as a crime committed by a man or men to kill a woman because of her gender, with hatred for her condition as a woman, punishable with thirty to forty years of prison (Decree 23 of 2013).^17^ The reform responded to international pressures to comply with conventions such as CEDAW (1979) and Belém do Pará (OEA 1994). The general considerations recognized that the state “is part of different international treaties to protect women” and that it was “obligated to protect and guarantee the prevention, investigation, and sanction of violence against women.” Penalties only applied when the perpetrator was in a relationship with the victim, the feminicide was preceded by domestic violence or sexual violence, or when the crime was committed with cruelty (Article 118A). The law lacked specific mechanisms for the prosecution of these crimes; it was largely symbolic and did not recognize gender violence against women. The lack of connection between the feminicide law and other forms of domestic violence weakened its punitive potential.

Honduras also has failed to align its VAW law with the judicial code. Since 1997, VAW laws stated the need for specialized jurisdictions in the courts; however, over two decades later, those changes in the judicial system have not been implemented. In 2010, Agreement No. 4 established gender-specialized courts within the justice system. This agreement recognized the need to “mainstream the gender perspective in planning and management institutes in the jurisdictional and administrative processes … to ensure greater access to justice” (CEDIJ 2017); however, the agreement never materialized in changes in the courts. Despite the approval of specialized courts^18^, greater access to justice remains an unfulfilled goal (INAM 2014, 51).

In 2014, the National Institute for Women (Instituto Nacional de la Mujer), in its *National Plan to Prevent Violence against Women*, affirmed that “violence against women is one of the first causes of complaints to the National Police and one of the leading causes of morbidity among women of reproductive age and therefore an important public health, human rights, and security issue” (INAM 2014, 13). Because the Law against Domestic Violence in Honduras also protects men, statistics of the national judicial system classify domestic violence cases by the complainant’s gender. Between 2012 and 2020, 12 percent of cases were filed by men (CEDIJ 2020). And because the law gives equal protection to all family members, women have been sentenced under this law.

In sum, VAW laws in Honduras have three fundamental problems at their inception. First, they are suffused with familyism ideologies and thus embedded in the state’s mandate to protect the family, marriage, maternity, and childhood, without a specific focus to protect women from violence. Second, punishments not classified in the penal code are sanctioned with community service, diminishing the seriousness of violent crimes against women. Since restitution is made to the community and not to the victim, reparations for domestic violence have lower stakes than the high costs of reporting and sentencing these cases. And third, because domestic violence law gives equal weight to every family member, women are classified as potential perpetrators of domestic violence who can be prosecuted under the law that aims to protect them. In this way, Decree 132 became a tool for protecting aggressors, giving them the right to resocialization and reincorporation into the household.

### De-escalated implementation: The case of Nicaragua

*De-escalated implementation* refers to the legal positioning that diminishes the regulation of VAW laws. In Nicaragua, familyism ideologies permeated the regulatory decree to protect and preserve the family unit—above women’s rights—by reincorporating mediation in domestic violence cases without changing the judicial system’s infrastructure. In this case, VAW laws agree with the penal code; however, the judicial system and its infrastructure do not protect women from domestic violence. The law is comprehensive, yet a lack of infrastructure in the courts (e.g., gender-specialized courts) and a regressive regulatory code undermine regulation and implementation. While the 2012 VAW law prohibited “mediation,” the regulatory decree of 2014 reincorporated mediation between husband and wife before imposing domestic violence charges against a partner. This change prioritizes the family unit and undermines women’s safety.

Nicaragua has three laws to protect women against violence. Law 230 of 1996, (“Reforms and additions to the Penal Code for the prevention and punishment of domestic violence”) defined domestic violence in the family and classified it as a crime.^19^ It included visible and invisible injuries and “any alteration in the health and any other damage to the physical or psychological integrity if these effects are produced by an external cause” (Law 230, Article 137). Nicaragua was one of the few countries with laws that specified penalties and prison times for violence against women in the region. Furthermore, although Law 230 was progressive, positioned favorably vis-à-vis the penal code, and stipulated the crimes and punishments associated with offenses against women, it failed to deter domestic violence. Reflecting the family’s prioritization, Law 230 applied to all family members and did not recognize women’s unequal position within the family, making them vulnerable to domestic violence.

Law 779 of 2012 (“Law on violence against women and reforms to the Penal Code”) acknowledged the failures of Law 230 of 1996.^20^ It stated that “the existing regulations to stop gender violence have not obtained the results sought for the effective protection of women’s life, freedom, and personal integrity” (Law 230, Preamble). The new law declared that “the state must respond to VAW and protect women’s human rights and stipulated the actions the state must undertake to guarantee women a life free of violence” (Article 3). The law described feminicide as a crime in which “a man, in the framework of unequal relationships of power between men and women, kills a woman either in public or private spheres” (Article 9). Furthermore, it defined the relationship between crimes regulated by the feminicide law and those regulated by the penal code (García-Del Moral and Neumann 2019; Neumann 2017). The law specified that the “prosecution of crimes established in the [VAW] Law should be governed by principles and procedure established in the ‘Criminal Procedure Code’ in the forms and terms listed for serious and less serious crimes as appropriate” (Article 39). However, the agreement of VAW law and penal code was problematic to implement because gender-based crimes must comply with the higher evidentiary standards of other crimes, making it difficult to provide evidence and prove instances of rape in a context where the “decorum” of the woman is at stake.

Law 779 suffered strong opposition leading to a reform (Decree 42 of 2014) that reversed its accomplishments. Regulatory Decree 42 of 2014 reincorporated the family as the fundamental unit of society, thus relegating women and gender violence to a secondary place.^21^ It inscribed the family as a priority for protection, as shown in the twenty-eight of its fifty-one articles that pertained to mediation as a mechanism to protect the family. Under the judicial system’s oversight, this statute included “prior mediation,” a step before the enforcement of penal actions.^22^ This change aligned with the Catholic Church’s argument that Law 779 attacked the family and religious values (O’Brien and Walsh 2020; Neumann 2017). As the feminist and women’s movement leader Azahálea Solís (2013, 1) stated: “the mediation mechanism does not work [to protect women against violence], it indulges and deliberately favors the perpetrators of the crime, therefore leaving the women defenseless.” Regulatory Decree 42 incorporated “family counseling” as a recourse for every family member. Despite recognizing the unequal relations between women and men, the decree strongly emphasized prevention and institutional family counseling within the community, delegating conflict resolutions to community-based organizations even in cases of violent crimes that legal authorities should handle.

The judicial system played a vital role in the approval of Decree 42 of 2014. The misalignment between VAW laws and judicial codes created the impression that lack of mediation was an obstacle to implementing the VAW law. After examining verdicts between 2012 and 2014, we found that the courts performed forty-one mediations (June 2012– September 2013) even when the law prohibited it (Article 46). During this period, the courts issued 1,131 dismissals, including charges of child support neglect (763), intimidation and threats against a woman (135), rape of a child fourteen years or younger (26), physical violence (21), sexual abuse (20), and rape (15), arguing that without mediation these cases could not be solved. Therefore, mediation was incorporated into Law 779 and has become regular practice (Cerda López 2014). While Law 779 was progressive on paper, mediation as a tool to keep families together in Nicaragua’s courtrooms violated Article 46. It derailed the implementation process, thus aligning with the idea that the family unit should be preserved through mediation, even at the expense of women’s safety.

### Abstracted standardization: The case of Guatemala

Another impeding legal positioning is abstracted standardization. Here the content of VAW laws agrees with the penal and judicial codes. However, because these codes take precedence, violence against women and feminicide cases are held to very high evidentiary standards requiring prosecutors to prove beyond reasonable doubt that the motive for a woman’s death was hatred based on gender (Ngozi, Iyioha, and Durojaye 2018). Despite recognizing the profoundly unequal power relations between women and men, which can result in the killings of women with impunity (Guatemala, Decreto no. 22–2008, Preamble), familyism ideologies infuse domestic violence cases, as women are required to resolve their differences and attend family mediation before bringing charges against an aggressor.

Guatemala has created three laws to protect women from gender violence. Decree 97 of 1996 contained articles on the prevention and punishment of domestic violence.^23^ The wording of Decree 97 allowed for different interpretations, and it was ambiguous about whether it could work in conjunction with other legal codes (Menjívar and Walsh 2016). Guatemala also approved Decree 7 in 1999, recognizing discrimination and violence against women.^24^ This law delineated state actions to safeguard women’s lives and created minimum protection mechanisms such as maternity leave and women’s freedom to select their spouse, last name after marriage, profession, and occupation. For the first time, a law highlighted the importance of women obtaining, acquiring, managing, and enjoying goods, property, and real estate to guarantee gender equality (Articles 12, 22, and 25). The objective was to modify judicial practices and customs that involved the persistence or tolerance of violence against women (Article 18). This VAW law showed the need for the “promotion of normative changes so that women subject to violence in any of its forms … have access to protection, prompt judicial and administrative mechanisms, to guarantee the compensation of damage and other means of reparation” (Article 18).

Third, in 2008 Guatemala approved Decree 22, “Law against femicide and other forms of violence against women,” which classified feminicide as violence against women.^25^ Feminicides are acts of violent killings of women in the context of unequal power relations between women and men. For the first time, VAW law agreed with the penal code. This agreement was a vital step because, even if prior VAW laws aimed at eradicating violence against women, they were ineffective if other “adjacent” laws disfavoring women (e.g., in marriage, divorce, alimony) remained intact (Menjívar and Walsh 2016). The 2008 feminicide law became a fundamental instrument for delineating the state’s obligations and creating exclusive jurisdiction to prosecute crimes against women. Most important, this law outlined a plan to transition from a nonregulatory state of the law to regulation with mechanisms to prosecute feminicides and protect domestic violence victims.

These changes to the law were necessary but not sufficient to guarantee women’s protection. Thus, in addition to changes in the penal code, VAW laws in Guatemala brought several judicial system changes. Before Decree 22 of 2008, domestic violence cases were reported to family mediation centers (Decree 97 of 1996, Article 4). In these instances, both parties allegedly reconciled and resolved their “differences” before pressing charges against one of the parties for domestic violence, child support, or alimony, for example. Between 1996 and 2008, victims were forced to negotiate with their aggressors for the family’s benefit, once again privileging familyism over women’s lives and rights. However, after 2008, Decree 22 (Articles 14–15) referred domestic violence cases to newly created specialized courts for criminal investigation.

After all these changes and despite efforts to implement VAW and feminicide laws, Guatemala’s judicial system claimed that it had not been able to reduce violence against women offenses because it was overwhelmed with the number of cases reported (UNHCR 2015). To solve this problem, the judicial system created Specialized Justice Units in 2010. However, the most significant problems in the implementation of VAW laws in Guatemala are “the difficulties of the judicial system staff as a whole to undertake the gender approach as a consolidated institutional culture, which is related to the persistence of macho prejudices and discrimination against women within the institution” (Contreras García 2016, 76). Thus, laws protecting women seem comprehensive on paper; however, in practice, due to the need for counseling from family mediation centers before formally filing charges, high evidentiary standards to prove wrongdoing in cases of feminicide, and lack of support from the courts, Guatemala’s laws impose significant obstacles to prosecute cases of violence against women.

### Delayed execution: The case of El Salvador

Finally, El Salvador constitutes a case of delayed execution. Here, misalignment of VAW laws with the penal code equips perpetrators of domestic violence or feminicide with mechanisms to contest or postpone their sentences, arguing that VAW laws violate their right to due process and presumption of innocence. Because of the lack of agreement, the penal code’s higher legal positioning prioritizes the defendant’s rights over any rights and protection of the victims. Consequently, the specialized judicial system applies protocols and procedures of the penal code that do not address the needs of domestic violence and feminicide cases.

Familyism ideologies seep through VAW laws in El Salvador. In 1996, the country approved Decree 902, a law against domestic violence, but it did not address violence against women.^26^ Instead, the law focused on equal rights for “men, women, sons, and daughters” (Decree 902, Article 2). The generic approach to violence against any family member ignores that women are the primary targets of domestic violence, followed by children, disabled individuals, and the elderly. The focus on all household members presumably having equal vulnerability to violence reveals the prioritization of the family to the detriment of women and the undermining of VAW laws’ aim to protect women. Furthermore, even though this law identified different forms of violence (i.e., psychological, physical, sexual, or economic), it did not include feminicide. Its main goal was to prevent acts of domestic violence without prejudice to criminal liability, which meant that the penal code and its ruling over women’s killings prevailed over Decree 902 (the law against domestic violence). Because of the lack of agreement between Decree 902 and the penal code, the superior laws supersede any rights and protections under VAW laws. Without the potential for substantive implementation, the law was symbolic and only made small advances to protect women. In this legal landscape, VAW laws were placed at a lower level, rendering them futile in one of the most violent contexts for women in the world.

In 2011, El Salvador approved Decree 520 (Special comprehensive law for a life free of violence for women).^27^ This law was the first statute to include women’s right to a life free of violence and discrimination. It recognized women’s right to education without stereo-typical behaviors or social and cultural practices based on inferiority and subordination (Article 2). The law declared that women had the right to physical, psychological, and moral integrity and equal protection under the law. A secularity principle was also introduced, preventing appeals to costumes, traditions, or religious considerations to justify violence against women. This addition is a critical feature as the devaluation of women is predicated on ideas that women are deserving of violence if they do not behave according to ideologies upholding familyism biases. Unlike Decree 902 of 1996, the 2011 law explicitly benefits women. It “presumes that the types and modalities of violence contemplated in the law originate from the unequal relationship of power or trust, in which the woman is at a disadvantage with respect to men” (Decree 520, Article 7).

One of the contributions of Decree 520 is the classification of feminicidal violence as an extreme form of gender violence both in public and private settings. This law included creating a specialized commission to guarantee the law’s application and development of related policies. However, one of the law’s main weaknesses is that the responsibilities, punishments, and prosecution procedures were left general and vague, underscoring its symbolic value. Ideas such as the right to education, identification of risk factors by police, and the responsibilities of educational institutions are essential, but the decree lacked concrete procedures to investigate domestic violence and feminicide cases. The law incorporated articles to extend financial resources to domestic violence victims and make financial resources available to implement the law. This law was more progressive than others in the region that lacked earmarked financial resources to sustain them. However, the alignment of the VAW laws and the penal code was left for future legislation, weakening the implementation of Decree 520, despite the financial components that came with this law.

Given the lack of agreement between VAW law and the penal code, the processes and sanctions of domestic violence and feminicide cases sustain endemic forms of violence against women and fail women in efforts to seek justice. The devalued position of women and prioritization of the family unit are expressed in the lack of arbitration and prosecution of feminicide cases. Despite social practices that devalue women, social class advantage or bureaucratic will may advance a case in some instances, but in the end there will be technical and organizational obstacles that block justice for feminicide cases. For example, defense attorneys delay their clients’ prosecution in highly publicized cases, alleging the defendant’s constitutional right to a psychiatric evaluation and hence a “constitutional right of defense.” Between 2011 and 2021, El Salvador reported 3,922 feminicide cases, 19,125 domestic violence cases, and 30,784 sexual assault cases (ORMUSA 2022), demonstrating that laws on the books, placed lower in the juridical structure, fall far short of protecting women on the ground.

## Discussion and conclusion

Honduras, Nicaragua, Guatemala, and El Salvador have passed an array of VAW and feminicide laws. Nevertheless, women continue to suffer physical abuse, sexual assaults, beatings, rapes, and killings and endure some of the region’s highest levels of gender violence. Why are these laws so ineffective? Given this region’s record-high levels of violence against women coupled with the existence of a range of laws meant to address this issue, the Central American case provides fruitful ground to examine why VAW laws have failed so remarkably to achieve their aim. Lessons from our examination can illuminate similar cases worldwide, as parallel legal structures and familyism ideologies inform legal processes elsewhere.

Failures in VAW laws implementation have been examined by explaining the gap between laws on the books and laws in practice. We build on this scholarship (Adamson, Menjívar, and Walsh 2020; Ghosh and Choudhuri 2011) by focusing on obstacles to implementation that arise from the lower placement of VAW laws vis-à-vis the penal and judicial codes. Thus, we extend the scholarship on the gap by taking a step back, before implementation, to the inception of VAW laws to examine their legal positioning, a fundamental step to understanding obstacles to VAW law implementation.

We use Bourdieu’s lens of symbolic violence (2001) to illuminate how familyism ideologies that disfavor women in social life seep through to shape VAW laws. We argue that VAW laws’ unequal positioning and status vis-à-vis penal and judicial codes debilitate them from the start and reflect the broader inequalities and disparities that women experience in society. Gendered inequalities and conceptions of women’s position in the larger social context permeate the juridical positionings and content of VAW laws to weaken their potential. Ideologies that prioritize the family unit to the detriment of women (Menjívar 2011) and equate the needs of women with those of other family members shape legal practices in the form of “familyism” (Velásquez Díaz Vargas Quintanilla, and Pérez Villanueva 2016; Facio 1992; Eichler 1988). Societal sexist practices predicated on family unity thus infiltrate judicial systems where other laws hold a higher status than VAW laws and create critical obstacles to implementation starting at the laws’ inception.

We identified four forms of legal positioning: undermined sanctions in Honduras, where VAW laws are not in agreement with the penal or judicial codes; de-scaled implementation in Nicaragua, where VAW laws are embedded with the penal code but not the judicial code; abstracted standardization in Guatemala, where VAW laws are in agreement with both the penal and judicial codes but high evidentiary standards prevent women from seeking justice; and finally, delayed execution in El Salvador, where VAW laws are not embedded in the penal code despite the development of specialized courts. Each of these legal positionings, paired with language in laws, prioritize family unity and undermine VAW laws’ implementation, reproducing the normalized lower place women occupy in society and law.

Despite multiple formal initiatives to protect women, the cases we presented exemplify how poor implementation of VAW laws conforms to traditional ideas of family and of women, which reproduce gender violence and render current legislation limited. The four-country comparison shows the diverse processes through which familyism permeates VAW laws and is refracted in the alignment and misalignment of VAW laws with the penal and judicial codes leading to faulty implementation. To be clear, not all countries experience the same levels of mismatch between these laws, but the variations in this mismatch reveal similar social contexts where women and their bodies are devalued and family unity prioritized.

What lesson do we glean from these cases for improving the prospects of effective VAW laws?^28^ No matter what legal actions are taken, if the social context that disfavors and devalues women does not change and gender inequalities become more entrenched, the outcomes will always be poor. If familyism ideologies take precedence, any legal initiative to protect women from violence will remain symbolic. Even with expanded state capacity to address the four obstacles we have examined, women’s access to justice would remain compromised and widespread violence and impunity rates high. Finally, although our examination focuses on the specifics of Central American juridical practices, we highlight the theoretical and analytical relevance our examination may have for other countries and contexts, including more affluent countries, where gaps in VAW laws’ implementation also exist and where women’s rights and protection take second place to concerns for family unity. Finally, our examination may be directly relevant to women’s and human rights organizations promoting policy changes on the ground.

## Footnotes

1 *Femicide* refers to the killings of women because they are women (Radford and Russell 1992); *feminicide* calls attention to the state’s role in these killings, directly or indirectly, through indifference or negligence (Lagarde y De los Ríos 2010). We use *feminicide* to highlight these state failures to protect women from gender-based violence.

2 In the literature, *familism* refers to the role of machismo in Latin American culture. However, we use *familyism* to highlight the role of family-first ideology in laws that supersede women’s protections, rights, and lives.

3 We do not mean to lump together different forms of violence against women; we know that feminicide, rape, and other assaults are distinct crimes and are necessarily addressed through different laws and penalties. In the interest of space and to keep the focus on our main points, we use this general term.

4 This includes all the laws on women’s rights and protections, for example, national laws that ratify international treaties, constitutional reforms, and VAW laws (Guatemala passed 26 laws, El Salvador 10, Honduras 12, and Nicaragua 7). Given space limitations, in the empirical sections of the article, we focus on the primary VAW laws only.

5 These numbers refer to feminicide cases in the courts, which are usually fewer than cases recorded by the morgue or police. No reliable statistics have been collected or published by Nicaraguan institutions since 2014.

6 These practices are not specific to Central America; similar issues can be found, in different guises and versions, throughout Latin America and around the world. A focus on Central American cases provides empirical ground to theorize further why laws meant to protect women fail them so consistently.

7 Evidentiary standards refer to evidence presented in court, which can be either a preponderance of evidence (in civil cases) or proof beyond reasonable doubt (in criminal cases). Feminicide cases prosecuted in criminal courts require that the victim (or her family) produce evidence such as witness testimony to show that the defendant committed the crime, and that no other explanation can be deduced.

8 The normative hierarchy is a rank ordering (or hierarchy) of the laws that constitute a legal system. Laws within the system have unequal positionings and weights (Shelton 2017). Implementing a law (e.g., VAW laws) cannot contradict another law (e.g., penal and judicial codes) that is above it (or superior to it) in the normative hierarchy. Examining the positioning of a law with respect to other laws shows the relative weight a law has vis-à-vis other norms and rules.

9 We use the terms *agreement* and *alignment* interchangeably to reference the equivalency, superiority, or inferiority between two laws.

10 Bourdieu (2001, 2) uses the notion of symbolic violence to refer to the “gentle violence, imperceptible and invisible even to its victims, exerted for the most part through the purely symbolic channels of communication and cognition (more precisely, misrecognition), recognition, or even feeling.”

11 Following Bernardes (1985, 290), familyism ideologies are founded and framed in religious texts. As the Central American cases conform to the doctrine of various churches (e.g., Catholic, evangelical), religious teachings profoundly mold understandings of what a family should look like. This important topic, however, is beyond the scope of this article.

12 “The identification of the woman-person with the woman-family is one of the manifestations of familyism. Although it appears natural that the woman is more linked to the family than the man, this does not imply that women do not have needs as a human person, which are not identical to the family’s needs. Family and woman are not synonymous” (Facio 1992, 96).

13 We use *laws* and *decrees* interchangeably. In the Central American cases in this study, laws created by the legislative branch are called decree laws; however, they are laws approved by legislative processes and not by presidential decree.

14 Historically domestic violence, alimony complaints, and other family issues have been regulated by the family code and resolved in family courts. With the emergence of VAW and feminicide laws, cases of violence against women have been transferred to civil or criminal courts. Therefore, VAW/feminicide laws claim the recognition of women as independent of their families. We address this critical issue in a separate article.

15 Honduras, Congreso Nacional, Decreto 132–1997, Ley contra la Violencia Doméstica, *Diario Oficial La Gaceta* no. 8, tomo 414, November 15, 1997.

16 Honduras, Congreso Nacional, Decreto 250–2005, Reformas a la Ley contra la Violencia Doméstica, *Diario Oficial La Gaceta* no. 30, tomo 950, March 11, 2006.

17 Honduras, Congreso Nacional, Decreto 23–2013, Reforma a los Artículos 27 y 321 del Decreto 144–83, Contentivo del Código Penal, *Diario Oficial La Gaceta* no. 33, tomo 92, April 6, 2013.

18 Honduras, Corte Suprema de Justicia, Acuerdo No. 04, Acuerdo para La Creación de las Unidades de Género del Poder Judicial, *Diario Oficial La Gaceta* no. 32, tomo 373, September 30, 2010.

19 Nicaragua, Asamblea Nacional de la República, Ley no. 230, Reformas y adiciones al Código Penal para Prevenir y sancionar la violencia intrafamiliar, *La Gaceta, Diario Oficial*, no. 191, 1996.

20 Nicaragua, Asamblea Nacional de la República, Ley no. 779, Ley Integral Contra la Violencia hacia las Mujeres y de Reformas a la Ley no. 641 “Código Penal,” *La Gaceta, Diario Oficial*, no. 35, 2012.

21 Nicaragua, Presidencia de la República, Reglamento a la Ley 779, Ley Integral Contra la Violencia hacia las Mujeres de Reformas a la Ley no. 641, Ley del “Código Penal,” *La Gaceta, Diario Oficial*, no. 143, 2014.

22 According to the National Police, the filing and document reception system (ORDICE) required proof of previous mediation records before filing criminal cases, imposing severe barriers for women to access justice. And when those records existed, there was no control over the implementation of protections or enforcement of mediation commitments (Nicaragua, Policía Nacional 2009, 24).

23 Guatemala, Congreso de la República, Decreto no. 97–1996, Ley para Prevenir, Sancionar y Erradicar la Violencia Intrafamiliar, *Diario de Centro América* 1996, no. 34.

24 Guatemala, Congreso de la República, Decreto no. 7–99, Ley de Dignificación y Promoción Integral de la Mujer, *Diario de Centro América* 1999, no. 29.

25 Guatemala, Congreso de la República, Decreto no. 22–2008, Ley contra el Femicidio y otras Formas de Violencia contra la Mujer, *Diario de Centro América* 2008, no. 27.

26 El Salvador, Poder Legislativo, Decreto 902, Ley contra la Violencia Intrafamiliar, *Diario Oficial* 1996, no. 241, tomo 333.

27 El Salvador, Poder Legislativo, Decreto 520, Ley Especial Integral para una Vida Libre de Violencia para las Mujeres, *Diario Oficial* 2011, no. 2, tomo 390.

28 We thank one of the reviewers for pointing us to this critical question.
